# Impact of heat stress on cow reproduction and fertility

**DOI:** 10.1093/af/vfy027

**Published:** 2018-11-10

**Authors:** David Wolfenson, Zvi Roth

**Affiliations:** Department of Animal Sciences, Faculty of Agriculture, Food and Environment, the Hebrew University, Rehovot, Israel

**Keywords:** cooling, cows, fertility, heat stress, ovarian function

**Implications**

- Summer heat stress is a major cause of low fertility in dairy cattle. Consequently, cows are unable to conceive.
- Severe hyperthermia results from high metabolic heat production and low rate of evaporative heat loss. Application of efficient cooling is a must to minimize heat stress.
- Multiple reproductive processes are impaired, including oocyte competence, embryonic growth, gonadotropin secretion, ovarian follicular growth steroidogenesis, development of the corpus luteum , and uterine endometrial responses.
- Treatments combined with cooling may improve fertility. Combinations of GnRH and PGF_2α_ are used to improve fertility. Embryo transfer and progesterone supplementation also improve fertility of subpopulations of cows.

## Introduction

### Hyperthermia in summer

Heat stress during the summer disrupts several reproductive processes, resulting in a pronounced depression of conception rate in dairy cows worldwide. The rise of internal body temperature during the summer is responsible for the impaired reproduction. A major cause for sustained hyperthermia during the summer is high milk production, which continues to rise. The processes of milk synthesis and secretion increase cows’ metabolic heat production. For instance, heat production of cows yielding 30 kg/day milk is twice as high as maintenance heat production of nonlactating cows, and that of high milk yielding cows giving 55 kg/day is about three times higher than maintenance heat production.

**Figure 1.**
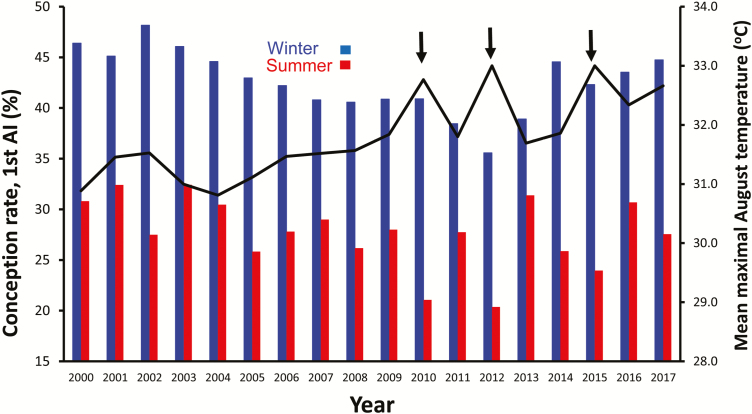
Conception rates in large dairy farms in Israel of cows first inseminated during the winter (January–March) or summer (July–September) of 2000–2017. Mean maximal air temperatures during August month of each year are presented as a black curve. Extreme summer conditions in 2010, 2012, and 2015 are associated with markedly lower conception rates. Adapted from Y. Lavon and E. Ezra, Israel Herd Book, with permission.

Maintenance of normal and constant body temperature requires a balance between endogenous heat produced in the body and the amount of heat lost from the body to the environment. When heat production exceeds heat loss, the body temperature rises. Body temperatures of high milk yielding cows located in a wet region were found to start rising exponentially at air temperatures of 26–27 °C. Thus, even a small rise in air temperature, on the order of 1–2 °C, due, for instance, to global warming, may induce severe hyperthermia in dairy cows. This is clearly seen in Figure 1, which demonstrates the depressive effect of summer heat on the conception rate of lactating cows artificially inseminated (**AI**) in the summer months over the last 18 years to as low as 27.7%, compared with 42.6% during the cool winter months. Moreover, the “slightly” more severe conditions during the summers of 2010, 2012, and 2015, about 1.5 °C above average summer air temperatures, further decreased conception by an additional 5% units (Figure 1).

### Cooling approaches

The need to use heat-abatement strategies is a result of high metabolic heat production due to high milk yield, but also of the low sweating rate in cows—about 1/4 to 1/3 of that in horses and man. Until the 1980s, cooling was based on blocking direct solar radiation and using ventilation; it did not involve spraying water on the cows. However, these basic means did not prevent hyperthermia, which led scientists in Israel to examine direct wetting of cows’ skin to facilitate evaporative cooling. This cooling approach is based on short-term spraying of water followed by its evaporation from the skin by air from fans (Flamenbaum et al., 1986; Berman and Wolfenson, 1992). The sprinkling and ventilation cooling system is commonly used today worldwide for dairy cows in hot/warm climate countries. Efficient cooling requires several cooling windows per day, consisting of cycles of water spraying and ventilation lasting about 30–50 min each. An alternative approach to cooling cows is low-profile cross-ventilation in free-stall buildings. This approach requires closed barns and is based on evaporative cooling of the microenvironment inside the barn. The low-profile cross-ventilation system is used mainly in the United States.

The efficiency of cooling in commercial farms can be conveniently compared by calculating the ratios between summer and winter milk production and conception rates. Calculations demonstrate that efficient cooling management in high ranking farms makes it possible to maintain milk production in the summer very close (98%) to that in winter. However, the ratios also indicate that summer conception reaches 68% of that in winter, much less than the value obtained for milk production. It is thus becoming clear that the reproductive system is highly susceptible to thermal stress.

The economic outcome of seasonal differences in fertility between summer and winter is significant, resulting from uneven milk production throughout the year: excess production in the winter and deficiency in the summer lead to high economic expenses. Furthermore, efforts to achieve a successful conception of cows in summer are also expensive because more AI is required per pregnancy. Other means that may improve conception in summer, based on various hormonal treatments, are described later in this review. Worth noting is that the use of cooling to prevent severe hyperthermia of cows and maintain the smallest possible rise in body temperature is a prerequisite for successful hormonal treatment.

**Figure 2.**
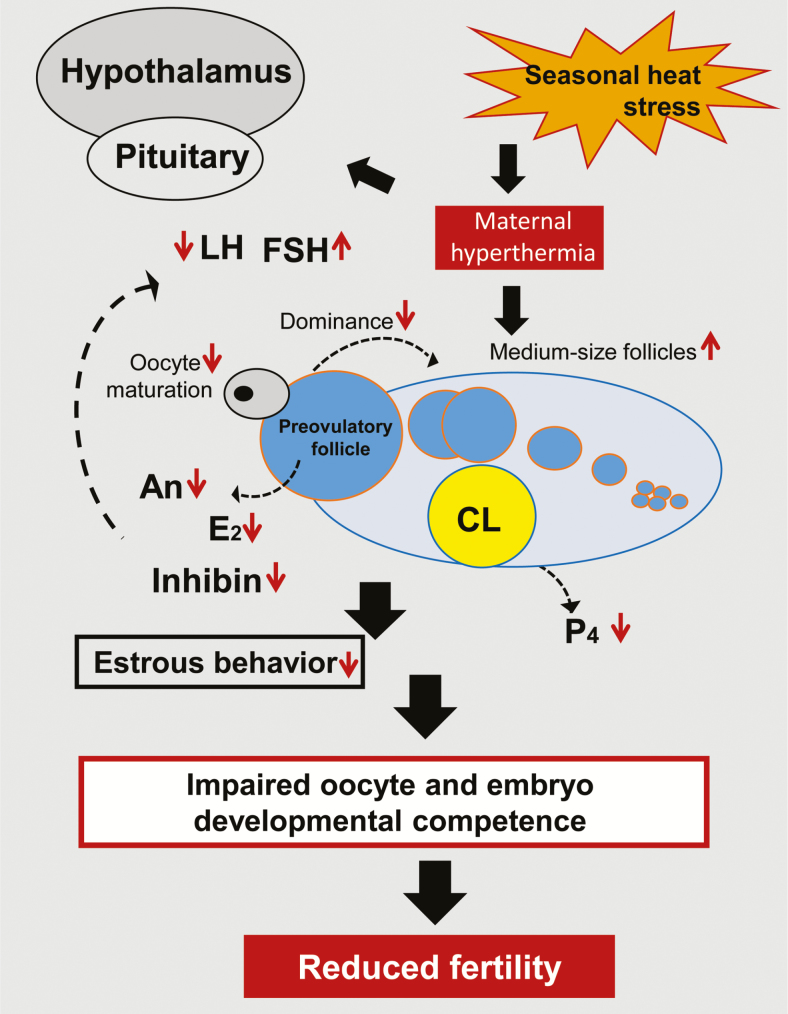
Diagram illustrating the long-term effects of seasonal heat stress on the hypothalamus-pituitary-ovarian axis and its involvement in reducing fertility of lactating cows. Reduced LH secretion is associated with reduced follicular estradiol (E_2_) secretion. Reduced dominance of the preovulatory follicle is reflected by reduced androstenedione (An) and E_2_ concentrations and is associated with reduced estrous behavior. Increased number of medium-size follicles (6–9 mm in diameter), most likely due to reduced dominance, is associated with reduced inhibin and increased FSH concentrations. Reduced oocyte and embryo developmental competence is associated with disruption of nuclear and cytoplasmic maturation. Reduced plasma progesterone (P_4_) concentration is related to impaired function of the CL. Reduced fertility in heat-stressed cows is presumed to result from additive effects. Adapted from Roth and Wolfenson (2016).

Here, we concentrate on the main processes that are impaired in female cattle and lead to low fertility. The overall scheme of heat stress-induced impairments of reproductive functions is presented in Figure 2. The disruptive effects of thermal stress on male reproduction are beyond the scope of this review.

## Gonadotropins

The gonadotropins luteinizing hormone (**LH**) and follicle-stimulating hormone (**FSH**) play important roles in ovarian function, including regulation of follicular growth, ovulation, and corpus luteum (**CL**) development. There is some discrepancy in the literature regarding gonadotropins, but most studies indicate that heat stress depresses LH secretion and compromises its function. For instance, follicle tissues obtained from heat-stressed cows were shown to secrete lower levels of steroids under gonadotropin stimulation (Bridges et al., 2005). Other studies showed lower concentrations of GnRH-induced LH surge under heat stress (Gilad et al., 1993). In another study, decreased expression of LH receptor was reported in the follicles of heat-stressed goats. Reduced LH surge and/or alteration in the sensitivity of follicular cells to LH might, in turn, impair the cascade of events leading to ovulation and formation of a functional CL. Moreover, reduced estradiol concentrations under heat stress in cows close to ovulation may also disrupt the preovulatory LH surge.

Unlike LH, FSH secretion increases under heat stress and is associated with a larger number of follicles growing in the ovaries (Wolfenson et al., 1995). In agreement with this, Roth et al. (2000) showed a pronounced decrease in plasma inhibin concentration in heat-stressed cows, which in turn caused an increase in plasma FSH concentration, known to stimulate follicle growth in the ovaries. These alterations might explain the significant rise of double ovulation and the marked rise in calving of twins following summer insemination.

Low LH surge may cause the development of suboptimal CL secreting low levels of progesterone. Together, altered gonadotropin secretion can depress cow fertility in the summer. A possible approach to “correcting” the situation is to administer a single dose of GnRH at the onset of estrus coinciding with the secretion of the low endogenous LH surge, consequently inducing a normal LH surge. Indeed, studies in which GnRH was administered at the onset of estrus (Kaim et al., 2003) significantly increased conception rates in heat-stressed cows. A single dose of GnRH analogue was administered 2–3 h after onset of estrus. Improvement of conception rate was noted mainly in cows with low body condition, known to have low LH surge. For unclear reasons, the improvement was also recorded in first calving cows, and it was much less pronounced in mature cows (Kaim et al., 2003).

## Ovarian Follicles

Cows usually exhibit two follicular waves in a 21-day estrous cycle. In each wave, a single follicle becomes largest and dominant, and the others become atretic and disappear. The dominant follicle of the second wave develops into the preovulatory follicle at the end of the cycle when the endocrine status “permits” induction of ovulation. Heat stress alters follicular growth dynamics in cows. Two physiologically significant impairments associated with attenuation of dominance are worth mentioning here. The first is a rise in the number of large follicles in a follicular wave, which probably underlies the increased numbers of twins following summer inseminations. The second is extended duration of dominance of the preovulatory follicle, resulting from its early emergence (Wolfenson et al., 1995). This finding might explain in part the negative impact of heat stress on fertility because the extended duration of the preovulatory follicle has been shown to be associated with depression of fertility.

**Figure 3.**
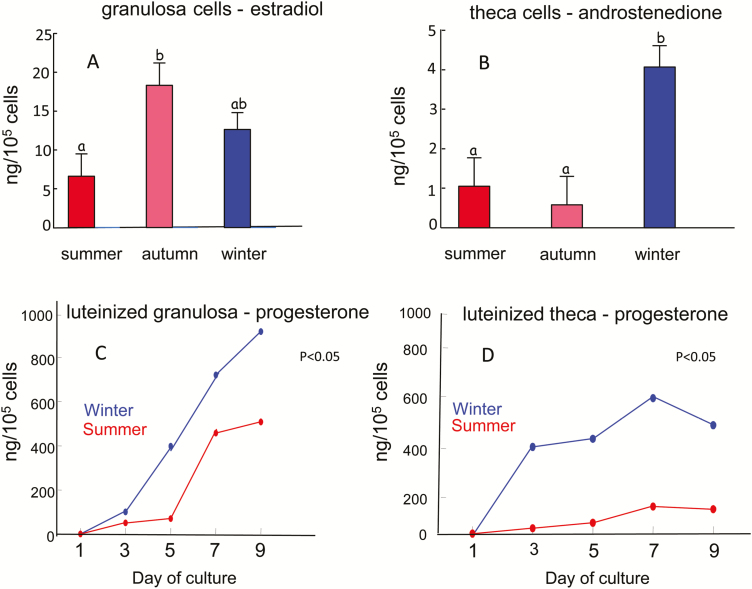
Seasonal differences in steroid production. (A and B) Estradiol production by granulosa cells (A) and androstenedione production by theca cells (B) obtained from dominant follicles on day 7 of the estrous cycle are lower in summer than in winter. (C and D) Progesterone production by luteinized granulosa (C) and theca (D) cells obtained from dominant follicles on day 6 of the cycle is lower in summer than in winter. Cells underwent differentiation to luteal cells for 9 days. Adapted from Wolfenson et al. (1997, 2002).

A reduction in the steroidogenic capacity of follicles under thermal stress is characterized by less aromatase activity of granulosa cells and decreased estradiol concentration in the dominant follicle (Wolfenson et al., 1997). Figure 3A and B demonstrates lower estradiol production by the granulosa cells in summer vs. autumn and winter, and lower androstenedione production by the theca cells in summer and autumn vs. winter. Seasonal (summer and subsequent autumn) or experimental (5 days of heat exposure in a hot chamber) heat stress had a carryover effect on steroid production (Roth et al., 2001b). Potentially adverse effects of low estradiol production might include impaired estrus duration and intensity; suppression of LH secretion which, in turn, might impair events associated with ovulation; development of ovarian cysts; and alteration of CL functioning, associated with reduced progesterone production (Wolfenson et al., 2000). With respect to depression of estrous behavior in the summer, use of Ovsynch and timed AI protocols have been shown to improve the overall pregnancy rate of cows in summer, most likely because, among other reasons, all of the cows are inseminated, regardless of estrus manifestation (de la Sota et al., 1998). Given the long-lasting effect of heat stress on ovarian follicles, various types of hormonal administration to stimulate follicular growth were tested. Induction of follicular cycles by repeated injections of GnRH and PGF_2α_ eliminated the disruptive effect of heat stress on follicular function. This approach was further developed to improve summer and autumn fertility (Friedman et al., 2011), as discussed further on.

## The Corpus Luteum

The CL secretes progesterone, which is essential for embryonic development. Luteal insufficiency refers to the status of a CL that does not secrete adequate amounts of progesterone to support pregnancy, and it has therefore long been associated with low fertility in cows and other female animals. Progesterone supplementation during early pregnancy under nonheat stress conditions improves reproduction to a certain extent; however, findings are controversial because not all studies show a benefit to fertility. Under the conditions of summer heat stress, provision of exogenous progesterone to increase suboptimal endogenous progesterone concentrations may improve conception rate, nevertheless, the benefit of this approach is controversial as well. Studies indicate that in most cases, exposing cows to short-term, acute heat stress is not associated with a reduction in progesterone concentration. The higher concentration of progesterone found in acute type studies has been related to adrenal secretion of progesterone or to the severity of the thermal stress (Bridges et al., 2005). In contrast, a significant decrease of progesterone is typically obtained when cows are exposed to long-term, chronic, seasonal heat stress (Wolfenson et al., 2002). This can be attributed to disruption of the process of CL formation, or to low synthesis of progesterone under hyperthermia, or may be a result of impaired preovulatory follicles which subsequently form a CL with suboptimal function (Wolfenson et al., 2002). The latter possibility is clearly shown in Figure 3C and D, where luteinized granulosa and theca cells obtained from follicles in the summer produced much less progesterone than their counterparts obtained in the winter.

A possible approach to increasing progesterone concentration after insemination in the summer is to insert Controlled Internal Drug Release (CIDR) containing progesterone for a period of 2 weeks, starting on day 5 ± 1 after AI. A prerequisite to obtaining a beneficial effect is efficient cooling, otherwise, embryos will not survive. A study by Friedman et al. (2012) showed that CIDR treatment increases conception rate by 6% (not significant); however, the treatment significantly increased the conception rate in subgroups of cows with low body condition after calving, and in cows that exhibited uterine disorders at parturition. Based on the latter, a follow-up study (O. Shiff et al., unpublished data) was conducted in which the CIDR was inserted on day 5 ± 1 after AI only in cows with low body condition after calving or cows diagnosed with uterine disease postpartum. Results confirmed the findings of the earlier study, showing improved conception rate in subgroups of treated cows in the summer. The reasons for the beneficial effect of exogenous progesterone on specific subgroups warrant further research.

## The Oocyte

**Figure 4.**
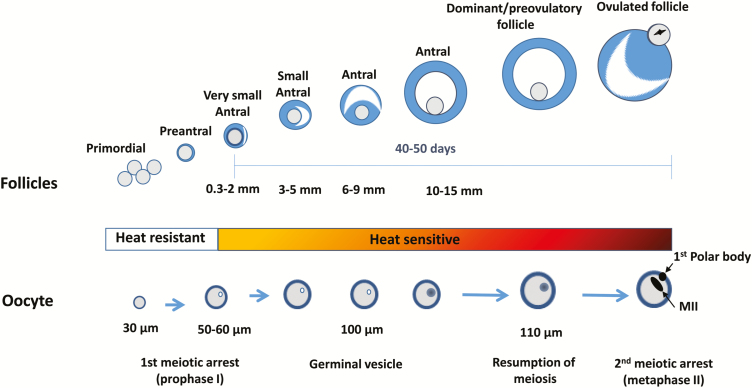
Diagram illustrating stage-dependent pattern of resistance/sensitivity of the ovarian pool of follicles and their enclosed oocytes to heat stress. The primordial, primary, and secondary follicles are heat-resistant, whereas the developing antral follicles, including the dominant and preovulatory follicles, are sensitive to heat exposure with a prominent effect on the germinal vesicle-stage oocyte (developing stage) and metaphase II (MII)-stage oocyte (ovulation). Adapted from Roth (2017).

The ovarian pool of oocytes is also sensitive to elevated temperature. A stage-dependent pattern of resistance and sensitivity to heat stress of the follicles and their enclosed oocytes are presented in Figure 4. The oocyte acquires its developmental potential in a stepwise manner during follicular development and therefore, heat stress-induced perturbations in follicular functioning can lead to reduced competence of its enclosed oocyte. Oocytes collected from Holstein cows during the summer exhibited a delay in the two first embryonic divisions (Gendelman et al., 2010). Other studies showed a reduced proportion of oocytes that were fertilized and further developed to the blastocyst stage under heat stress. A period of two to three estrous cycles was found to be required for recovery from summer heat damage and appearance of competent oocytes in the subsequent autumn (Roth et al., 2001a), indicating a long-lasting effect of heat stress on the ovarian pool of oocytes. This might explain the reduced fertility during the autumn, when cows are not exposed to environmental thermal stress. It should be noted that only a subpopulation of the ovarian follicles, rather than the entire follicular reservoir, is damaged upon maternal hyperthermia, reflected by spontaneous recovery of oocyte competence and conception rate during the autumn and subsequent winter. In light of this, enhanced removal of impaired follicles has been suggested to improve fertility (Roth et al., 2001a). In particular, three consecutive follicular waves induced by GnRH and PGF_2α_ during the summer and autumn improved conception rate, mainly in first calving cows and cows with high body condition score postpartum (Friedman et al., 2011). The authors believe that incorporating this approach of enhanced removal of impaired follicles, in subpopulations of cows, will improve, to some extent, the fertility of dairy cows during the summer and autumn.

The mechanism by which heat stress affects the oocytes involves cellular and molecular impairments. Exposing oocytes to heat shock during maturation impaired the rearrangement of their microtubules and microfilaments (Roth and Hansen, 2005), and a high proportion of heat-stressed oocytes were arrested at the metaphase I (MI) stage and had a damaged spindle apparatus. Heat shock of 41 °C reduced the proportion of germinal vesicle stage oocytes that resumed meiosis and progressed to the metaphase II (MII) stage (Payton et al., 2004). Taken together, heat stress-induced alterations in nuclear maturation might be associated with fertilization failure.

A seasonal comparison of mitochondrial distribution indicated a high proportion of category I (i.e., mature) oocytes in the winter, a low proportion in the summer, and an intermediate percentage in the autumn (Gendelman and Roth, 2012). Two potential mechanisms associated with mitochondrial function—apoptosis and oxidative stress—have been documented (for review, see Roth, 2017). Exposing oocytes to 41 °C during maturation increased the proportion of oocytes with fragmented DNA. The expression of apoptotic genes was higher in repeat breeder cows during the summer (Ferreira et al., 2016). Oxidative stress was also suggested to be involved in hyperthermia-disrupted fertility. Exposure of oocytes to heat shock during in vitro maturation increased reactive oxygen species (**ROS**) and reduced the ability of the oocyte to cleave and develop into a blastocyst. Antioxidants have been suggested to overcome the adverse effects of heat stress. For instance, the antioxidant epigallocatechin gallate, the most abundant flavonoid component of green tea, increased the proportion of fertilized oocytes and the percentage of blastocysts in heat-stressed mice.

Heat-induced impairments in maternal transcripts have been shown to underlie the response of the oocyte to heat stress, with further consequences in the developing embryo. Comparison of oocytes collected during the summer and winter revealed differential expression of maternal transcripts (*C-MOS*, *GDF9*, *POU5F1*, and *GAPDH*) involved in oocyte maturation and early embryonic development (Gendelman et al., 2010). The most prominent seasonal variation was a reduction in *POU5F1* mRNA expression in the hot season. Another seasonal study reported the lower expression of genes associated with oocyte maturation (*FGF16*, *GDF9*) in cows during the summer (Ferreira et al., 2016). Another publication reported differential expression of *Cx43*, *DNMT1*, and *HSPA14* in embryos developed from oocytes collected during the summer, relative to those collected during the winter (Pavani et al. 2016).

## The Embryo

While much of the effect of heat stress involves alterations in the follicle and its enclosed oocyte, preimplantation embryos are also sensitive to elevated temperature, in a stage-dependent manner (Hansen, 2007). Two-cell stage embryos are more sensitive to heat stress than those at four- and eight-cell stages. Embryos at later developmental stages (i.e., morula, blastocyst) are more resistant to heat stress (Hansen, 2007). Interestingly, heat shock differentially affects embryonic development in different breeds, with a moderate negative effect in *Bos indicus* (Brahman and Nelore) and a larger negative effect in *Bos taurus* (Angus, Holstein).

The mechanism underlying the embryo’s acquisition of thermotolerance seems to be associated with changes in the balance between free radical generation and antioxidant protection. In vitro administration of antioxidants (such as anthocyanin and dithiothreitol) protected embryos from heat shock (Sakatani et al., 2007). On the other hand, supplementation of vitamin E, known to have antioxidant capability, failed to improve bovine embryos’ tolerance to heat shock. Similarly, supplementation of vitamins A and C did not have any beneficial effect in heat-stressed cows. Treatment of dairy cows with melatonin, a potent ROS scavenger, in the summer before calving, improved their reproductive performance in the subsequent lactation (Garcia-Ispierto et al., 2013).

The balance between pro- and antiapoptotic factors plays an important role in embryonic survival. In cattle, apoptosis does not occur until the 8- to 16-cell stage embryo. Inhibition of heat-induced apoptosis by a specific caspase inhibitor improved embryonic survival. In agreement with this, insulin like growth factor 1 (IGF-I) administration to in vitro-derived embryos improved their resistance to heat shock (Jousan and Hansen, 2007). However, treatment of lactating cows with bovine somatotropin to increase IGF-I concentration did not have any positive effect on pregnancy rate during the summer.

Given that preimplantation embryos at early stages of development are highly sensitive to heat stress, embryo transfer at day 8, to bypass the thermosensitive developmental stages, has been suggested (Hansen, 2013). Embryo transfer during the summer increased pregnancy rate to those achieved with AI or embryo transfer in the winter. It is worth noting that pregnancy rate following embryo transfer can be compromised when the recipient cows cannot maintain normothermia (Vasconcelos et al., 2006), suggesting that the extent of the blastocyst’s thermotolerance is limited.

## Conclusions

The reproductive tract, in particular, the ovarian components (i.e., follicles, oocytes, CL), and preimplantation embryos are highly sensitive to elevated temperatures. The authors believe that using an efficient cooling system to maintain normothermia in cows is a prerequisite to any additional remedial approach; body temperature of the recipient cows is critical during embryo transfer; hormonal treatments to support CL function and embryonic survival are more efficient if the cow maintains normal body temperature. Given that the effect of heat stress on fertility is multifactorial in nature, a combination of treatment approaches might be most effective.

## About the Authors

**David Wolfenson** is professor emeritus at the Faculty of Agriculture, Food and Environment of the Hebrew University in Israel. He specializes in reproduction and environmental physiology of domestic animals. His research interests focus on impacts of heat stress and disease-related stress on ovarian function, and strategies to improve fertility of dairy cows.

**Zvi Roth** is associate professor at the Department Animal Science, the Faculty of Agriculture, the Hebrew University of Jerusalem, Israel. He leads a Reproductive Physiology and Embryology group which studies the effect of environmental stress on oocyte competence and embryonic development in cattle. Stressors include endocrine disrupting chemicals, food-borne toxins, and thermal stress.
